# Hyperbaric Oxygen Therapy Dampens Inflammatory Cytokine Production and Does Not Worsen the Cardiac Function and Oxidative State of Diabetic Rats

**DOI:** 10.3390/antiox8120607

**Published:** 2019-11-30

**Authors:** Rita Benkő, Zsuzsanna Miklós, Viktor Antal Ágoston, Katrine Ihonvien, Csaba Répás, Roland Csépányi-Kömi, Margit Kerék, Nóra Judit Béres, Eszter Mária Horváth

**Affiliations:** 1 Department of Physiology, Semmelweis University, Budapest 1094, Hungary; csepanyi-komi.roland@med.semmelweis-univ.hu (R.C.-K.); horvath.eszter@med.semmelweis-univ.hu (E.M.H.); 2 Institute of Clinical Experimental Research, Semmelweis University, Budapest 1094, Hungary; miklos.zsuzsanna@med.semmelweis-univ.hu (Z.M.); ikatrine89@gmail.com (K.I.); repas.csaba2@mentok.hu (C.R.); nagy.margit@med.semmelweis-univ.hu (M.K.); 3 Clinical Toxicology and Emergency Department, Peterfy Hospital, Budapest 1076, Hungary; agoston.viktor@gmail.com; 4 Department of Pediatrics, Semmelweis University, Budapest 1083, Hungary; bnora1988@gmail.com

**Keywords:** diabetes, HBOT, oxidative stress, PARP, endothelial dependent relaxation, cardiac ultrasound, rat

## Abstract

Hyperbaric oxygen therapy (HBOT) is frequently used after soft tissue injuries and in diabetic patients with ulcerated wounds; however, its ability to increase oxidative stress casts doubts. Diabetes (DM) in male Wistar rats (*N* = 20) weighing 300 g were induced by a single dose of streptozotocin. Ten diabetics (DMHBOT) and 10 controls (CHBOT) underwent a one-hour long hyperbaric oxygen treatment protocol (2.5 bar) 12 times after the 3rd week of diabetes. Ten animals remained untreated. Eight weeks after diabetes induction, we measured the 24-hour blood glucose profile and cardiovascular function (sonocardiography and the relaxation ability of aortae). Malonyl-dialdehyde (MDA) and cytokine levels were measured in blood plasma. Poly(ADP-ribose) polymerase (PARP) activity was estimated in cardiac and aortic tissue. HBOT did not alter most of the cardiovascular parameters. PARylation in cardiac and aortic tissues, plasma MDA levels were elevated in diabetic rats. HBOT prevented the increase of MDA in diabetic animals. In addition, levels of the pro-inflammatory cytokine-induced neutrophil chemoattractant-1 (CINC-1) the levels of anti-inflammatory tissue inhibitor of metalloproteases-1 were not altered in diabetes or in hyperoxia. Our results suggest that HBOT does not increase long-term oxidative stress, and, similar to training, the TBARS products, nitrotyrosine formation and poly(ADP-ribosyl)ation may be eased as a result of hyperoxia.

## 1. Introduction

Hyperbaric oxygen therapy (HBOT) is a frequently applied remedy for soft tissue injuries [1] and it seems to be an effective way of enhancing wound healing in diabetic patients [2]. As a result of HBOT, plasma oxygen content and tissue oxygenation get into the normal range even in the depth of ulcerated wounds. HBOT also mobilizes stem cells, such as endothelial progenitor cells, therefore, may represent a therapeutic aid for revascularization [3]. However, as available oxygen content increases in the core of the body, cardiovascular oxidative damage may emerge as a possible consequence of HBOT [4,5]. 

Elevated oxidative-nitrative stress can be detected in diabetic patients and alternative glucose metabolic pathways emerge. As a result, inflammatory processes are initiated and a further increment of oxidative-nitrative stress is detectable too. All these processes contribute to increasing the probability of cardiovascular damage. Therefore the possible adverse effects of hyperbaric oxygen therapy (HBOT) on oxidative balance and consequently on the cardiovascular system in diabetes raise questions [5]. 

The effects of hyperoxia on cardiovascular parameters and cytokine production are contradicting. Elevation of reactive species formation during HBOT is proven in animal models and human studies [6,7,8] but it has not been established yet whether this elevation is preserved days or even weeks after the cessation of HBOT. The concern is that elevated reactive radical formation may lead to the worsening of chronic inflammation and cardiovascular status. According to the metanalysis of de Smet et al., the short-term outcome of HBOT is a significant reduction of wound area; however, the research team warns us about the possible long-term side effects due to increased oxidative stress [9]. On the other hand, HBOT has a hormetic effect that may lead to increased antioxidant capacity. It is sought that HBOT reduces neutrophil-endothelial adhesion [10] through inhibiting neutrophil recruitment; also, matrix metalloproteinase activation is abated [11]. Therefore, it is possible that the net result of HBOT is decreased inflammation and consequently improved cardiovascular function [12].

Poly(ADP-ribosyl)ation (PARylation) is a ubiquitous consequence of emerging oxidative and nitrative stress. Superoxide reacts with nitric oxide (NO), resulting in peroxynitrite (ONOO^−^) formation and the produced reactive species attack DNA, causing single- and double-stranded breaks. These breaks are recognized by poly(ADP-ribose) polymerase 1 (PARP), that builds up long, branching chains of poly(ADP-ribose) (PAR) from NAD^+^. The subsequent loss of NAD^+^ leads to ATP depletion and cell death. Lower but steady PARylation of nuclear proteins may help DNA repair but also increase NF-κB expression and cytokine production; therefore constant PARP activation leads to chronic inflammation too. Increased cytokine production activates immune cells and keeps up oxidative and nitrative stress, leading to a vicious cycle [13,14]. 

Previously it was shown that PARylation increased following hyperbaric oxygen treatment [15] only when 50 atm of oxygen pressure was applied. In a rat model of severe acute pancreatitis, HBOT along with PARP-inhibition had an additive effect [16]; however, long-term consequences of HBOT on PARylation has not been assessed before. 

In the presented study, we estimated the effect of HBOT in a rat model of fully developed type1 diabetes on the cardiovascular system, cytokine production, oxidative stress and subsequent PARylation. 

## 2. Materials and Methods

All investigations conform to the Guide for the Care and Use of Laboratory Animals published by the National Institutes of Health (NIH Publication No. 85–23, Revised 1985) and was approved by Veterinary and Food Administration Institute of Budapest (1896/003/204).

A total of forty male Wistar rats weighing around 300 g were used for the experiment. They had water and standard rat chow ad libitum during the course of the experiment. They were randomly distributed into four groups, 10 rats per group, as follows—no diabetes and no HBO treatment (Control); HBO treatment without diabetes (Control HBOT); Diabetes without HBO treatment (DM); and finally diabetes with HBO treatment as well (DM HBOT). Up to two animals per group deceased between the 3rd and 6th weeks of the experiment. 

**Figure 1:**
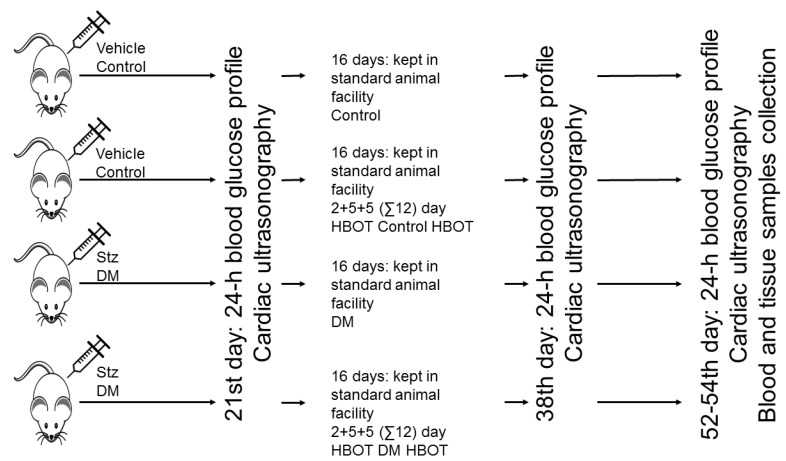
Forty rats were assigned randomly for receiving Streptozotocin (Stz) 70 mg/kg intravenously or vehicle. Three weeks later, the blood glucose and the cardiac functions of the animals were measured before undergoing hyperbaric oxygen therapy (HBOT, 2.5 bars, 60 min/day, on Thursday and Friday on the first week and the second and third week from Monday to Friday). At the end of HBOT the blood glucose and the cardiac functions of the animals were measured again. Two weeks later the rats were reassessed and killed under anesthesia to collect blood and tissue samples.

Twenty rats (future DM and DM HBOT) were injected intravenously with 70 mg/kg streptozotocin dissolved in citrate buffer (both Sigma Aldrich, St. Louis, MO, USA), under thiopentone sodium (Euthasol, Phylaxia-Sanofi, Hungary) anesthesia. Three weeks after the initiation of diabetes 24-hour blood glucose profile was taken using capillary blood samples obtained from the tail in every six hours from 8 AM. Ten control and 10 diabetic rats were enrolled in a daily hyperbaric oxygen treatment (Control HBOT) regimen 3 weeks after diabetes induction. HBO exposure was set at a pressure of 2.5 Bar for 60 min (the appropriate pressure was attained in 5 min, depressurization took an additional 8 min, this 13-minute period is not included in the 60-minute treatment period) on Thursday and Friday on the first week and the second and third week from Monday to Friday (12 days in the course of 16 days, no treatment on weekends; as it was commonly applied in human patients at Baromedical Ltd. (Budapest, Hungary; a Hungarian medical company providing HBOT) to decrease the risk of pulmonary side effects, Figure 1).

The day after the completion of the full HBO treatment protocol twenty-four-hour blood glucose profile of the rats was taken again.

To assess the long-term effects of HBOT, two weeks after the completion of the HBOT regimen 24-hour blood glucose profile and HbA1c levels were measured and the animals underwent echocardiographic measurement under halothane anesthesia (both M-mode and two-dimensional mode, 7–15 MHz, Hewlett Packard Sonos 5500, San José, CA, USA), end-diastolic and stroke volume (EDV and SV), ejection fraction (EF) and fractional shortening (FS) were calculated. Afterward, we sacrificed the rats in order to perform *ex vivo* measurements and sample collection (blood, whole hearts and thoracic aortae). The thoracic aorta was cleared from the surrounding periadventitial fat and was cut into 3–4 mm wide rings, laid in organ baths that was filled with warmed (37 °C) and oxygenated (95% O_2_, 5% CO_2_) Krebs’ solution (CaCl_2_ 1.6 mM; MgSO_4_ 1.17 mM; NaCl 130 mM; NaHCO_3_ 14.9 mM; KCl 4.7 mM; KH_2_PO_4_ 1.18 mM; Glucose 11 mM). Isometric tension was measured using isometric transducers (DMT, Hinnerup, Denmark) and digitized, stored and displayed (Biopac, Goleta, CA, USA) on a personal computer. A basal tension of 15 mN was applied and the rings were equilibrated for 60 min, vascular contractility was determined by phenylephrine dose-response curves (Phe, 10^−9^ to 3 × 10^−4^ M). The rings were allowed to equilibrate and to restore basal tone for 60 min. Afterward, phenylephrine precontraction (10^−6^ M) was induced and relaxation ability was determined by acetylcholine dose-response curve (Ach, 10^−9^ to 3 × 10^−4^ M) after). From each experimental group, 5 to 6 pairs of rings were gained and used during this experiment.

Heparinized and clotted whole blood samples were used for plasma and serum collection, respectively. The heparinized blood samples were gradient centrifugated on Histopaque-1083 (Sigma Aldrich, St. Louis, MO, USA) to isolate mononuclear blood cells, which were smeared on frosted glass microscopic slide and fixed in methanol. Intact aortic segments and hearts were fixed in 4% formaldehyde solution and then paraffin-embedded sections were cut. 

After deparaffinization of the sections, antigen retrieval (80 °C for 15 min in 0.1 M citrate buffer, pH 3) and blocking of endogenous peroxidase activity, the samples were incubated overnight at 4 °C with monoclonal anti poly(ADP-ribose) antibody (PAR, made in mouse, Tulip Biolabs, West Point, PA, USA, 1:500) or monoclonal anti poly (ADP-ribose) polymerase (PARP) antibody (Cell Signaling Technology, Danvers, MA, USA, 1:100). Secondary labeling was achieved using a biotinylated anti-mouse horse antibody (Vector Laboratories, Burlingame, CA, USA) (30 min, room temperature). Horseradish peroxidase-conjugated avidin (ABC kit, 30 min, room temperature, Vector Laboratories, Burlingame, CA, USA) and nickel-enhanced diaminobenzidine (6 min, room temperature, Vector Laboratories, Burlingame, CA, USA) were used. Tissue sections were counterstained with nuclear fast red for PAR (Reanal, Budapest, Hungary) or hematoxylin (PARP). To assess staining intensity, we captured 5 microscopic fields (at 200-fold magnification) of each sample and the percentage of the dye-positive area was determined in either ventricular wall area (cardiac samples) or endothelial cell layer area (aortae) by a blinded experimenter. Image analysis was done by ImageJ (1.49v NIH, Bethesda, MD, USA), the background of the original photos was subtracted, if present, debris was erased only from the background of the samples and a 2-bit conversion was applied. The threshold was set to the same value in case of every photo to measure positive and total area. No other manipulation of the pictures was done after taking the micrographs. The presented representative sample micrographs were taken at 400-fold magnification and the composite of the eight pictures was manipulated by applying a (common) histogram adjustment layer to achieve an evenly white background and acceptable contrast. 

For Western blot analysis, cardiac tissue was ground in liquid nitrogen with a mortar and pestle and suspended in ice-cold lysing solution (30 mM Na-HEPES, 100 mM NaCl, 2% (*w/v*) Triton-X-100, 20 mM NaF, 1mM Na-EGTA, 1 mM Na-EDTA, 100 mM benzamidine, 0.02% (*w/v*) diisopropyl phosphorofluoridate, 1% (*w/v*) aprotinine, 1% (*w/v*) protease inhibitor cocktail (Sigma-Aldrich), 1% (*w/v*) phosphatase inhibitor cocktail 2 (Sigma-Aldrich, San José, MO, USA) and 1% (*w/v*) phenyl methyl sulfonyl fluoride; pH 7.5) at a ratio of 250 μL of buffer per 0.5 g tissue (wet mass). Equal amounts of protein (50 μg) per lane were subjected to 10% (*w/v*) SDS-polyacrylamide gels and transferred to nitrocellulose membranes (GE Healthcare, Chicago, IL, USA) at 100 mA/cm^2^ for 2 h. After blocking with 5% (*w/v*) blotting grade (Bio-Rad Laboratories, Hercules, CA, USA) dissolved in PBS, supplemented with 0.1% (*w/v*) Tween 20, membranes were decorated with monoclonal anti poly (ADP-ribose) polymerase (PARP) antibody (Cell Signaling Technology, Danvers, MA, USA) overnight at 4 °C in 1:1000 dilution. Bound antibody was detected with enhanced chemiluminescence using horseradish peroxidase-conjugated anti-rabbit-Ig (from donkey) secondary antibody (GE Healthcare, Chicago, IL, USA) used in 1:5000 dilution. ImageJ software (NIH, Bethesda, MD, USA) was used for densitometry analysis. PARP was normalized against total protein performed by densitometry of Ponceau S stained membranes.

Systemic oxidative stress was gauged by malonyl-dialdehyde assay. MDA content of the plasma was detected based on the thiobarbituric acid reactive substances (TBARS) assay. The MDA-TBA product formed by the reaction of MDA and thiobarbituric acid (TBA) under high temperature (90–100 °C) and acidic conditions was measured at 540 nm [8].

To identify the cytokines involved rat cytokine array was performed (R&D Systems, Minneapolis, MN, USA) on plasma samples of a representative rat from each group (based on blood glucose levels, heart functions and vascular reactivity). After visual evaluation with the naked eye, the most affected cytokines-cytokine-induced neutrophil chemoattractant-1 (CINC-1), tissue inhibitor of metalloproteinases-1 (TIMP-1) and lipopolysaccharide-inducible CXC chemokine (LIX) were measured from each collected sample using commercially available ELISA kits according to the users’ manual (R&D Systems, Minneapolis, MN, USA). 

Results are reported as mean ± SEM. To investigate Gaussian distribution, D’Agostino and Pearson’s omnibus normality test was performed. In case of non-Gaussian distribution, logarithmic transformations were performed. This transformation resulted in normal distribution for CINC-1 and LIX levels. Statistical significance between two measurements was determined by the two-tailed unpaired Student’s *t*-test (HbA_1C_) and among groups, it was determined by two-way analysis of variance (ANOVA) with Tukey’s post hoc test. In case of differing variances between groups (area under glucose curve, MDA, CINC-1 and TIMP), nonparametric test (Kruskal-Wallis) was also applied with Dunn’s post hoc test. Probability values of *p* ≤ 0.05 were considered significant.

## 3. Results

**Figure 2:**
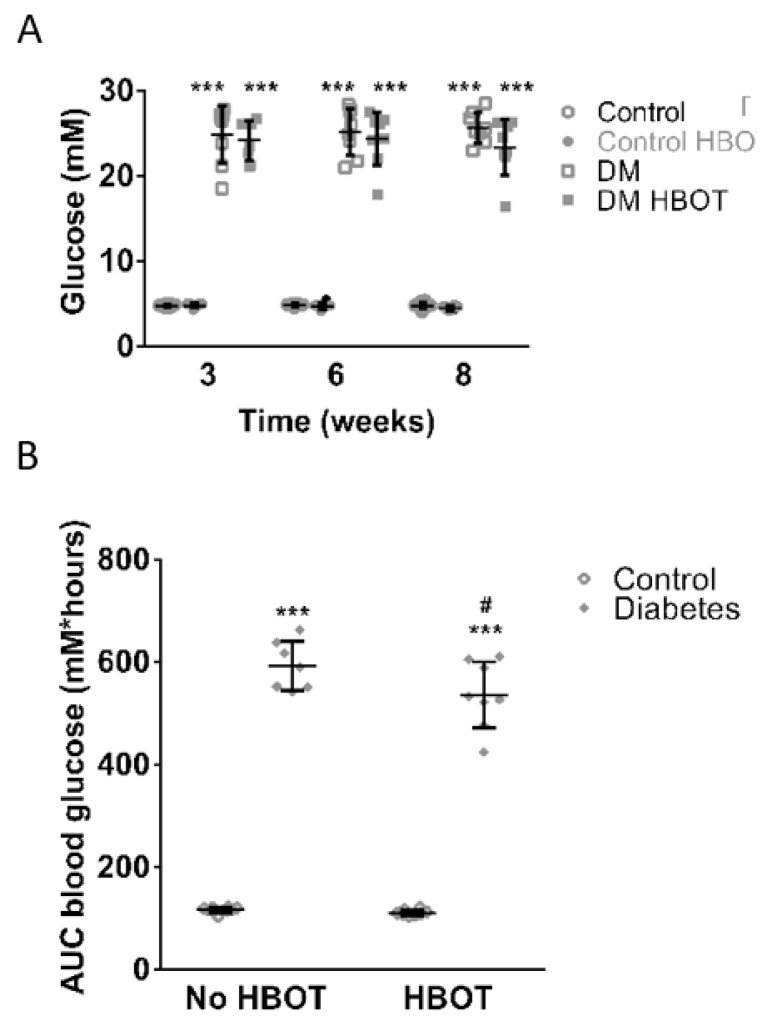
Carbohydrate household of control and diabetic rats with or without hyperbaric oxygen treatment (HBOT). (**A**) represents blood glucose levels of each group those 3, 6 and 8 (i.e., 2 weeks after the end of HBOT) weeks after the induction of diabetes HBOT had no effect on blood glucose levels: control animals (*N* = 9) and nondiabetic rats undergoing HBOT (Control HBOT, *N* = 9) had similar values; also diabetic rats without (DM, *N* = 8) and with HBOT (DM HBOT, *N* = 8) did not differ. On the other hand, both diabetic groups had significantly elevated blood glucose levels compared to the nondiabetic animals (Repeated measures ANOVA, *** *p* ≤ 0.001). (**B**) Area under the glucose curve (AUC) of all four groups 8 weeks after the induction of diabetes; each data point represents 24-hour area under the glucose curve. The HBOT did not alter the results in nondiabetic animals. Both diabetic groups had significantly elevated AUC glucose compared to the nondiabetic animals (Kruskal-Wallis with Dunn’s post hoc test, *** *p* ≤ 0.001). However, DM HBOT rats had significantly lower AUC than DM rats (Kruskal-Wallis with Dunn’s post hoc test, # *p* ≤ 0.05 DM v.s. DM HBOT). Data are presented as individual data points with mean ± SD.

Mean blood sugar levels (Figure 2) were significantly elevated in both diabetic groups 3 weeks after the induction of diabetes mellitus. Right after the completion of the HBOT regimen, there was no significant difference between the two diabetic groups. However, weeks later the effect of HBO sessions on the blood glucose level of the DM HBOT group was traceable. The area under the glucose curve was significantly smaller in the DM HBOT group compared to the DM group. No significant differences were observed between the non-diabetic groups. The levels of HbA_1_C were elevated in the two diabetic groups, whereas in non-diabetic rats HbA1c levels were under the detectable limit of 4%. HBOT caused a tendentious decrease in the HbA1c levels of diabetic rats, although it was below significance (DM: 8.26 ± 0.18% v.s. DM HBOT: 7.75 ± 0.24%; *p* = 0.115). 

**Figure 3:**
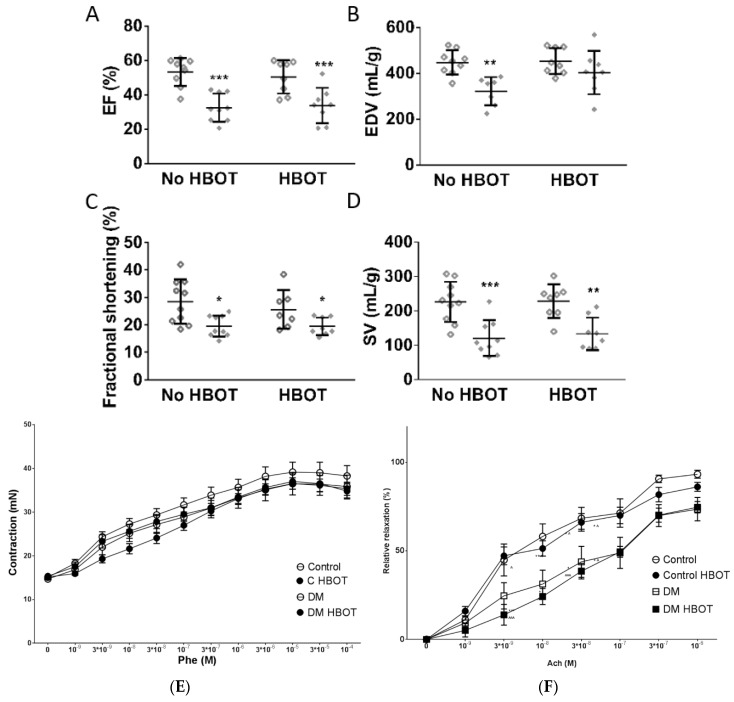
Cardiovascular performance of control and diabetic rats with or without hyperbaric oxygen treatment (HBOT) 8 weeks after diabetes induction. Stroke volume (SV) and end-diastolic volume (EDV) were normalized to left ventricular weight estimated by ultrasound examination. No cardiovascular parameter was altered by HBOT in healthy control animals. EF (**A**), FS (**C**) and SV (**D**) were significantly decreased in all diabetic animals (DM and DM HBOT, represented with solid diamonds) compared to control groups (non-diabetic animals are repreented with hollow diamonds). End-diastolic volume was also significantly deteriorated in DM rats but HBOT restored ventricular filling (DM HBOT do not differ from any other group) (**B**). (Two-way ANOVA, * *p* ≤ 0.05, ** *p* ≤ 0.01, *** *p* ≤ 0.001 vs. Control). Aortic contractility was not affected by diabetes or HBOT, as phenylephrine dose-response curves were identical in all groups. (**E**) but acetylcholine induced relaxation were corrupted equally in both diabetic groups. (**F**). Vascular reactivity is seemingly not influenced by hyperbaric treatment. Data are presented as with mean ± SEM for each vasoactive compound concentrations. (Repeated measures ANOVA, * *p* ≤ 0.05, ** *p* ≤ 0.01, *** *p*≤0.001 v.s. Control; **^˄^***p* ≤ 0.05, **^˄˄^***p* ≤ 0.01 **^˄˄˄^***p* ≤ 0.001 v.s. HBOT, Phe: phenylephrine, Ach: acetylcholine).

The cardiac parameters at the 8th week of diabetes were altered in all diabetic rats. Ejection fraction, stroke volume and fractional shortening were significantly lower in both diabetic groups; however, end diastolic volume was only reduced in the not treated diabetic animals. HBOT did not influence the cardiac parameters of non-diabetic rats. However, HBO-treated diabetic animals showed less worsening of EDV compared to non-treated diabetics (Figure 3). HBOT did not alter ejection fraction, stroke volume or fractional shortening measured under anesthesia. 

Phenylepinephrine induced contraction of aortic rings was not influenced by any treatment (Figure 3). Acetylcholine induced relaxation ability of the aortae deteriorated in DM rats compared to non-diabetic controls. There was no detectable difference between endothelium-mediated relaxation between HBO-treated and non-treated animals (Figure 3). 

**Figure 4:**
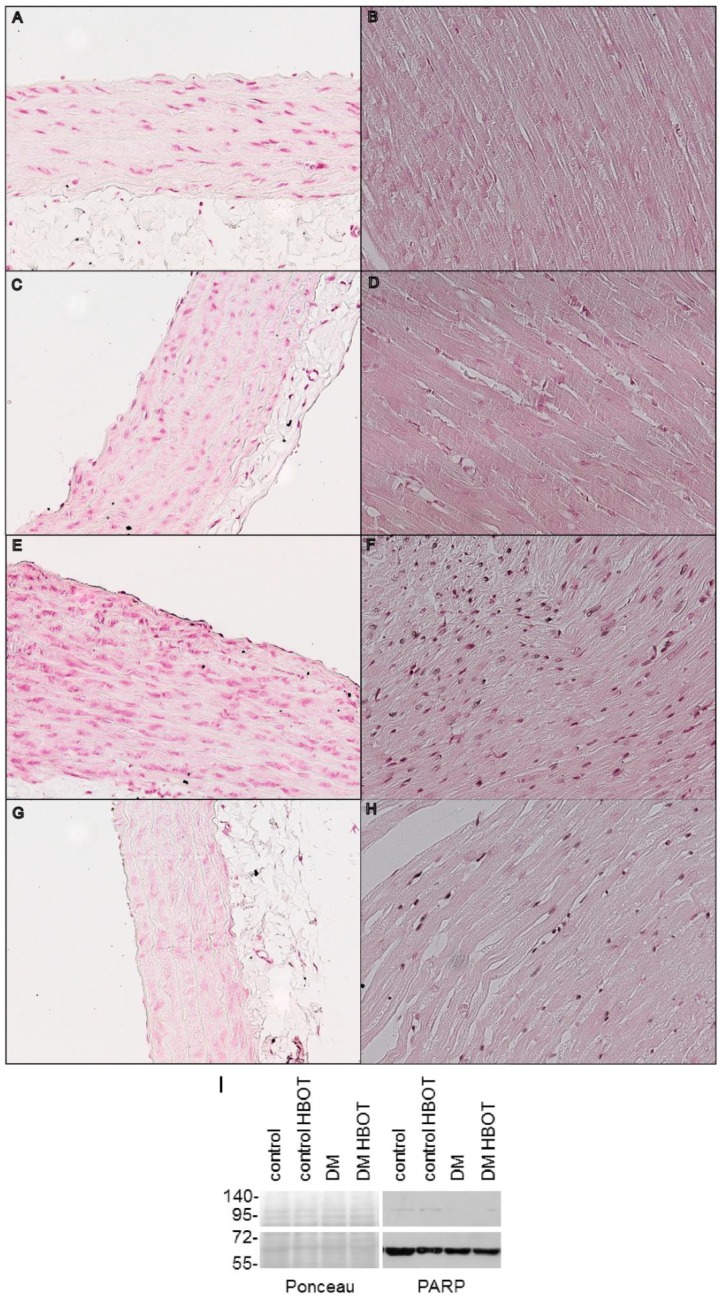

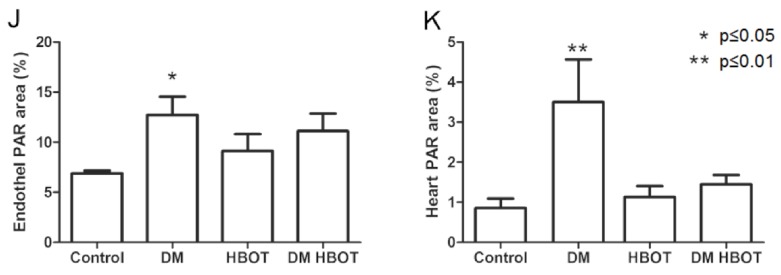
Poly(ADP-ribose) immunostaining of cardiac and aortic tissues of control and diabetic rats with or without hyperbaric oxygen treatment (HBOT). Representative immunostained sections are shown in light microscope images, at twentyfold magnification. Rows are the groups in the following order: up row Control (**A**,**B**), second row: Control HBOT (**C**,**D**), third row: DM (**E**,**F**), fourth row DM HBOT (**G**,**H**). Left column: aortic segments (**A**,**C**,**E**,**G**), right columns: left ventricular wall samples (**B**,**D**,**F**,**H**). Black precipitates in cardiac muscle and aortic endothelium are labelled poly(ADP-ribose) polymers. Photos were taken with 400-fold magnification. Original .bmp photos were resized (50%), copied to a common canvas forming a composite and vector layers were created for the framing lines and text. A histogram adjustment layer (gamma:1.29, whites clipped at 236, midtones expanded with -16 units; Corel PaintShop Pro X7) was added to the composite. (**I**) Poly (ADP-ribose) polymerase 1. in the cardiac samples assessed by Western blot. There was no difference in the enzyme expression. Cleaved PARP1 is visible at 70 kDa, full length PARP1 at 116 kDa. (**J**) Poly(ADP-ribose) immunostaining positivity of aortic endothelial layer. Protein PARylation was significantly increased in the untreated diabetic rats compared to controls, however HBOT prevented this elevation. (**K**) Poly(ADP-ribose) immunostaining positivity of the left ventricular wall of the heart. Similarly to the vascular endothelial layer, diabetic animals showed increased PARylation of the cardiac tissue, that was inhibited by HBOT. Data are presented as individual data points with mean ± SD. (Two-way ANOVA, * *p* ≤ 0.05 vs. Control; ** *p* ≤ 0.01 vs. Control).

The poly(ADP-ribosyl)ation of cardiac walls and aortic endothelia were elevated in untreated diabetic animals. Peculiarly, PAR staining remained unaltered in the cardiovascular system of non-diabetic rats and HBOT treatment prevented the increase of tissue protein PARylation in diabetic rats. (Figure 4) To assess whether PARP-1 expression or activity was altered as the result of diabetes and hyperbaric oxygen, protein levels of PARP-1 were estimated by Western blot analysis (heart) and immunohistochemistry (aortic endothel). There was no detectable change in PARP expression on the protein level in aortic endothel or cardiac tissue.

**Figure 5:**
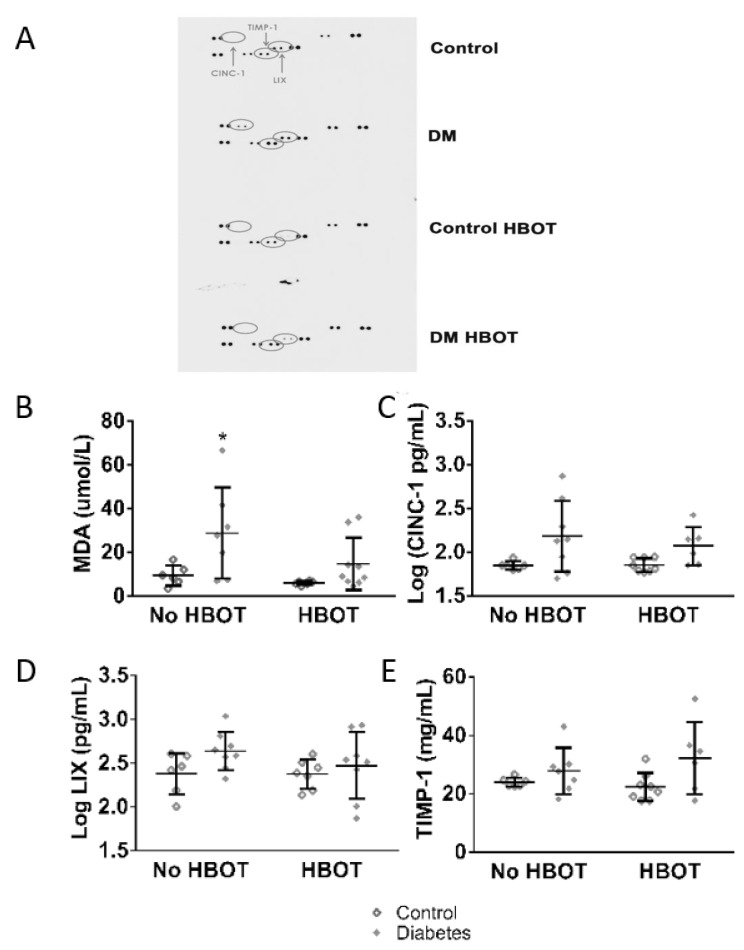
Malonyl-dialdehyde and cytokine levels of control and diabetic rats with or without hyperbaric oxygen treatment (HBOT). (**A**) depicts the method to estimate which cytokines are affected. Rat cytokine array was performed on one representative plasma sample of each group. The chosen cytokines were the proinflammatory CINC-1, LIX and matrix metalloproteinase inhibitor (anti-inflammatory) TIMP-1. These markers were further investigated in plasma samples from 6 to 8 animals per each group by ELISA assay. On (**B**), plasma MDA levels are visualized. DM rats had significantly higher levels of MDA than HBOT control. HBOT did not affect MDA levels in healthy rats but decreased MDA levels in DM HBOT rats. (**C**–**E**) shows the results of ELISA measurements of the selected cytokines. The pro-inflammatory cytokine CINC-1 levels (**C**) showed only increased variability in diabetic rats, LIX levels (**D**) was similar in all experimental groups. The matrix metalloproteinase inhibitor TIMP-1 levels (**E**) were low in each group. On panels **B**–**E**, data are presented as individual data points with mean ± SD. (Kruskal-Wallis with Dunn’s post hoc test * *p* ≤ 0.05 v.s. Control HBOT).

Without HBOT, plasma MDA levels were higher in diabetic rats compared to controls that underwent HBOT; this difference was attenuated in DM HBOT rats. The plasma levels of pro-inflammatory cytokines CINC-1 levels were sensitive to diabetes and hyperbaric oxygen (*p* = 0.0167); however, between-group statistical differences could not be determined. The anti-inflammatory TIMP-1 was not altered by diabetes in the lack or presence of HBOT (DM vs. DM HBOT *p* = 0.0214) (Figure 5). 

## 4. Discussion

Our aim was to assess the long-term consequences of hyperbaric oxygen treatment on cardiovascular and redox status of type 1 diabetic rats. In our study, we used a widely accepted animal model. Cardiac parameters of the diabetic rats were significantly worse than those of the controls, which suggests the presence of diabetic cardiomyopathy. Moreover, the deterioration of NO-mediated relaxing ability of aortae indicates that endothelial dysfunction also developed. Oxidative status, PAR-ylation and inflammatory signals were also elevated compared to control animals. These manifestations of diabetic symptoms are well-known and counted toward the major contributors of diabetic complications. 

Carbohydrate household and cardiovascular status of control rats undergoing hyperbaric sessions were not affected by the hyperoxia and their TBARS and inflammatory cytokine production did not show any alteration either. This suggests that non-diabetic patients are safe in this regard under the hyperoxic environment. Furthermore, it is sought that the development of type 1. diabetes mellitus can be postponed by HBOT, as it was shown in an animal model of autoimmune diabetes.

The study revealed that HBOT did not increase, however slightly decreased the blood glucose levels of diabetic animals, shown by the reduction of AUC glucose. Many previous studies found that HBOT leads to an elevation of blood glucose levels of streptozotocin-induced diabetic rats. First, it was thought that this elevation that follows augmented oxidative stress is a direct consequence of hyperoxia in a diabetic system [17,18]. For instance, Matsunami et al. described an elevation of blood glucose level parallel with severe alterations in the expression of antioxidant enzymes under HBOT in a rat model of streptozotocin-induced diabetes, concluding that HBOT further damages carbohydrate household and increases oxidative stress [18,19,20]. Later it was recognized that streptozotocin treatment itself causes a vast elevation of reactive oxygen species as part of its mechanism of action [21]. Therefore, one may speculate that streptozotocin-induced and naturally developing diabetes may react differently to HBOT. This hypothesis is supported by Faleo et al., who described a slower development of type 1. diabetes in non-obese autoimmune diabetic mice [22]. To prevent the confounding effect of streptozotocin treatment, our team decided to start hyperbaric sessions after the complete degradation/excretion of streptozotocin. Interestingly, other observations of type 2 human subjects suggested that carbohydrate household may be improved by HBOT [23]. Accordingly, we also observed decreased AUC glucose and a tendency of decrement in HbA1c in our model but—possibly due to the short timeframe of our study—it did not reach the level of significance. 

Our study also showed that HBOT did not deteriorate the cardiovascular status of diabetic animals; however, oxidative stress, poly(ADP-ribozyl)ation and inflammatory processes were dulled. 

Subclinical inflammation is present in diabetic patients. One typical executor of the chronic inflammation is PARP activity leading to elevated inflammatory cytokine (e.g., TNF alpha) production and consequential NF-κB expression. Poly(ADP-ribose) polymerase 1 (PARP) is activated as a response to DNA breakage in elevated oxidative stress. This increment of inflammatory transmitters leads to a further increment of free radical formation. Also, PARP plays a role in arresting or initiating cell death, promoting repair mechanisms and cooperating with transcription factor formation (reviewed by References [24,25]. As poly(ADP-ribosyl)ation was only elevated in untreated diabetic rats, our results suggest that hyperoxia indirectly inhibits PARP activation and the consequent inflammatory vicious circle. As the muted TBARS product suggests, we can hypothesize that the link between lower PAR-ylation and hyperbaric treatment may possibly be the activation of antioxidant mechanisms, as similar protection was achieved by Ayvaz et al. in a bile duct ligation model [26]. The suggestion that hyperbaric oxygen treatment and inhibited poly(ADP-ribosyl)ation cooperate in conserving tissue integrity comes from Inal et al. [16] who proved that oxidative stress parameters and histopathology are ameliorated by PARP inhibition and HBOT but their effects are increased when acting together in severe acute pancreatitis. In their model, acute pancreatitis caused a loss of superoxide-dismutase and glutathione peroxidase along with an elevation in MDA levels. Induction of pancreatitis was followed by two sessions of HBOT and data were collected after two sessions and two days. Interestingly, according to their findings HBOT acutely decreased oxidative stress, increased antioxidant capacity and protected tissue functions. According to our results, this theory can be applied to diabetes as well. In addition, inhibition of PARP resulted in a similar protection in acute pancreatitis; therefore, it was suggested that diabetic rats benefit from PARP inhibitors (e.g., References [27,28,29]).

Our group assessed endothelial function in the thoracic aortae. We accepted the assumption that peripheral vessels, mainly microcirculatory units, are similarly affected by hyperglycemia. One possible explanation to the worsening of endothelial function of hyperglycemic patients is the uncontrolled glucose uptake of endothelial cells and consequential mitochondrial overproduction of oxidative agents [30]. The role of skeletal muscle oxygenation in metabolic control is also influential. Ameliorated skeletal muscle oxygen supply described by Yamakoshi et al. [31] can be one reason behind improved inflammatory status and metabolic changes. In comparison, effects of training may have similar influence than that of HBOT on cardiovascular and inflammatory status of diabetic patients, because physical activity also elevates immediate oxidative stress and activates antioxidant mechanisms [32]. 

Increased activity of matrix metalloproteinases (MMPs), along with elevated oxidative stress, is proposed to be one of the leading causes behind the higher risk of ischemic events in diabetic patients. In a HBOT preconditioning model, it was shown that hyperbaric environment leads to a decrement of MMP-2 and 9 in an animal stroke model [33]. Also, previously, HBOT was proven to inhibit NF-κB expression in healthy rats. Due to the low number of samples and high variances of CINC-1 and TIMP-1 levels in the diabetic animals, our results do not allow to reinforce these observations; nonetheless, the presented findings do not point toward an increment in inflammatory processes after HBOT. Further inquiries may reveal a cohort, which could benefit HBOT as an anti-inflammatory intervention.

Cardiac dysfunction developing in maltreated diabetes can be the result of insufficient oxygen supply by the coronary circulation, subclinical systemic inflammation and elevated oxidative-nitrative stress and an increment of alternative metabolic pathway byproducts (recently reviewed by Reference [34]). Diastolic dysfunction develops earlier than systolic dysfunction. As hyperbaric treatment ameliorated the cardiac oxygen supply and alleviated inflammatory cytokine production, this could have been one reason of maintained diastolic filling. Regulation of cardiac function better maintained in HBOT animals due to the slower deterioration of autonomic functions [31].

Recently, it was shown that HBOT increased oxygenation in injured muscle tissue up to 24 hours after hyperbaric exposure, along with a reduction in edema formation. Better oxygenation of the affected tissues may contribute to the healing of the ulcerated wound. Furthermore, activation of the pro-inflammatory IL-6/STAT3 pathway is sought to participate in the inflammation following soft tissue injury. The peak of IL-6 production was detected prior in HBOT animals than in non-treated rats leading to earlier STAT3 phosphorylation. According to the team of Yagishita, STAT3 phosphorylation is a major step in satellite cell activation and tissue repair. Whether in diabetic patients, the progenitor cell activation contributes toward wound healing and conservation of cardiovascular status, needs to be further assessed [35].

## 5. Conclusions

As the present study was carried out on a group of healthy rats as well as on rats with increased cardiovascular risk and chronic subclinical inflammation, we can estimate that athletes, who typically have better cardiopulmonary health, do not develop more severe complications than diabetic patients while undergoing HBOT; therefore, similar results should be replicated in human athletes and patients with type 1 diabetes, then diabetic athletes who suffered sport-related injury might also undergo hyperbaric oxygen therapy. Likewise, diabetic patients may be treated with hyperbaric oxygen without significantly elevating their risk of worsening cardiac status. To summarize our findings, the results are in line with most of recent findings, that hyperbaric treatment may rather decrease oxidative stress in different conditions. This decrement is obviously detectable in our diabetic model, too. To assess whether this hypothesis is viable in humans, further observational approaches are needed. On the other hand, alterations of carbohydrate household did not reach the level of significance as a response to HBOT, possibly due to the short-term experimental settings. Correlations between cardiovascular status and tissue poly(ADP-ribozyl)ation were diminished in HBOT diabetic rats, because PARP activity was reduced without significant alterations in endothelial and heart functions. Considering the decreased pro-inflammatory LIX and CINC-1 expression, this may mean that HBOT breaks the vicious cycle formed by subclinical inflammation, oxidative stress and PARP activation.

## Abbreviations

Ach	acetylcholine
CINC-1	cytokine-induced neutrophil chemoattractant-1
DM	diabetes mellitus;
EDV	end diastolic volume
EF	ejection fraction
FS	fractional shortening
HBOT	Hyperbaric oxygen therapy
LIX	lipopolysaccharide-inducible
CXC	chemokine
MDA	malonyl-dialdehyde
MMP	matrix metalloproteinase
NO	nitric oxide
ONOO	peroxynitrite
PAR	poly(ADP-ribose)
PARP	poly(ADP-ribose) polymerase
Phe	phenylephrine
ROS	reactive oxygen species
SV	stroke volume
TBA	thiobarbituric acid
TBARS	thiobarbituric acid reactive substances
TIMP-1	tissue inhibitor of metalloproteases-1
